# Progress in understanding how clock genes regulate aging and associated metabolic processes

**DOI:** 10.3389/fphys.2025.1654369

**Published:** 2025-09-23

**Authors:** Yanhong Su, Meng Wang, Juan Chen, Yanan Bao, Ruikang Wen, Hui-Wen Ren, Zhi-Lin Luan

**Affiliations:** ^1^ Key Laboratory of Sports Human Science in Liaoning Province, College of Physical Education, Liaoning Normal University, Dalian, China; ^2^ Advanced Institute for Medical Sciences, Dalian Medical University, Dalian, China

**Keywords:** circadian rhythms, clock genes, aging, metabolic disorders, metabolic syndrome

## Abstract

The circadian system, primarily governed by the suprachiasmatic nucleus of the hypothalamus, consists of a central clock and peripheral clocks distributed across various body tissues. Clock genes generate a 24-h oscillatory cycle via a transcription-translation feedback loop (TTFL). Emerging evidence has identified circadian rhythm disruption as a significant contributor to the risk of metabolic disorders. With aging, the function of circadian rhythms declines, leading to metabolic dysfunction in multiple organs. This article reviews the molecular mechanisms underlying circadian rhythm disruption during aging, with a focus on telomere homeostasis, SIRT1-mediated epigenetic regulation, and the NAD^+^ metabolic pathway, and systematically analyzes the characteristics of rhythm imbalance in different metabolic organs. A comprehensive understanding of the correlation between circadian rhythms and aging is essential for developing strategies to combat aging and metabolic diseases.

## 1 Introduction

Biological rhythms represent significant time-based patterns formed by living organisms in response to Earth’s rotational and orbital changes. The circadian rhythm, a fundamental biological rhythm derived from the Latin terms “circa” (around) and “dies” (day), refers to the approximately 24-h rhythmic fluctuations in physiological and behavioral processes of life (Hastings et al., 2023). Biological clocks often used interchangeably with circadian rhythms, play a crucial role in regulating metabolic balance. Research indicates that over half of the protein-coding genes exhibit circadian rhythmicity. These internal rhythms, or biological clocks, are vital for maintaining metabolic balance, with research revealing that more than 50% of protein-coding genes display circadian rhythms (Zhang et al., 2014; Mure et al., 2018).

Mammalian circadian rhythms are regulated by a hierarchical network of clock systems. At the apex is the hypothalamic central clock located in the suprachiasmatic nucleus (SCN), which receives light information from the retina and controls peripheral clocks via neuroendocrine pathways, establishing a coordinated physiological rhythm network (Honma, 2018). This mechanism is driven by the transcription-translation feedback loop (TTFL) at a molecular level. The groundbreaking discovery of *PERIOD* gene mutants in *Drosophila* by Konopka and Benzer in 1971 marked the dawn of circadian gene research (Konopka and Benzer, 1971). The first circadian mutant mice were identified through a mouse behavioral screening in 1994 (Vitaterna et al., 1994), followed by the cloning of the mammalian circadian gene, *Clock* in 1997 (King et al., 1997). These milestones sparked a surge in studies on mammalian circadian mechanisms, leading to the identification of multiple new genes in the core clock loop (Tei et al., 1997; Gekakis et al., 1998; Kume et al., 1999; van der Horst et al., 1999; Vitaterna et al., 1999; Zheng et al., 1999; Bunger et al., 2000; Bae et al., 2001). Decade-long research has since revealed a complex regulatory framework, including non-coding RNAs (Takahashi, 2017; Abe et al., 2022).

Recent lifestyle changes driven by globalization have markedly disrupted human circadian rhythms. Epidemiological evidence shows that shift workers have a 40% increased risk of developing type 2 diabetes (Leproult et al., 2014) and a 23% higher susceptibility to cardiovascular disease (Su et al., 2021). In 2023, a large-scale cohort study published in a *Nature* sub-journal revealed a strong link between irregular sleep-wake cycles and elevated all-cause mortality (Shah et al., 2023).

A major concern is that as the global population ages, the decline in circadian rhythm functionality during aging has become a critical public health concern. Studies have shown that aging is accompanied by significant changes in sleep patterns, characterized by disruptions in sleep-wake cycles such as advanced sleep onset, fragmented sleep, and reduced sleep quality, which are closely associated with cognitive decline and metabolic syndrome (Banerjee and Ray, 2023).

Accumulating evidence highlights the association between circadian disruption and metabolic imbalances in aging, including obesity, insulin resistance, type 2 diabetes, and cardiometabolic disorders (Thaiss et al., 2014; Corrales et al., 2019; Orihara et al., 2020; Ding et al., 2021; Su et al., 2021). Unfortunately, modern human lifestyles pose challenges to maintaining metabolic homeostasis. For example, frequent consumption of high-calorie diets and constant caloric repletion blunt metabolic oscillations. Circadian interruption in shift workers has resulted in reduced insulin sensitivity and increased susceptibility to obesity and diabetes (Leproult et al., 2014). Skeletal muscle, the body’s largest metabolic organ, relies on circadian regulation to maintain glucose balance, mitochondrial function, and muscle regenerative capacity. Circadian disruption also affects osteoblast differentiation and inflammatory responses, thereby influencing the metabolic health of the musculoskeletal system. Thus, preserving circadian rhythm homeostasis is critical for maintaining metabolic health and mitigating the escalating pandemic of metabolic disorders (O'Neill and O'Driscoll, 2015). While research indicates that the central nervous system’s pivotal role in circadian regulation (Kim et al., 2020; Ding et al., 2021), the mechanisms governing peripheral circadian rhythms and their dysregulation in pathophysiological remain poorly understood.

A bidirectional regulatory relationship exists between aging and circadian rhythms (Stowe and McClung, 2023). On one hand, aging impairs the central clock’s ability to synchronize, as demonstrated by a greater than 50% reduction in SCN neuron firing (Verma et al., 2023). On the other hand, persistent circadian rhythm disruption may accelerate aging via epigenetic mechanisms (Osum and Serakinci, 2020). Recent key discoveries include: identifying a crucial circadian pattern in telomere length that declines with aging (Park et al., 2019; Osum and Serakinci, 2020); elucidating that SIRT1 controls multiple clock proteins by altering deacetylation, forming a metabolic-epigenetic loop, and contributing to cell aging (Amano et al., 2019; Osum and Serakinci, 2020; Sousa and Mendes, 2022; Kuatov et al., 2024); and uncovering that NAD^+^ metabolic dysfunction serves as a key link between circadian disruption and age-related metabolic disorders (Amano et al., 2019). These findings provide new insights into understanding metabolic disorders in aging.

A comprehensive understanding of the interplay among circadian rhythms, aging, and metabolism is essential for developing strategies to combat aging and metabolic diseases. Various circadian-targeted pharmaceuticals, such as REV-ERB agonists and SIRT1 activators, have advanced to clinical trials (Nohara et al., 2020). The article aims to systematically review the molecular mechanisms underlying circadian rhythm disruption during aging and their effects on distinct metabolic organs. The focus of this review encompasses recent advances in the study of clock gene regulatory networks, the roles of key components (e.g., SIRT1 and NAD^+^) in aging, the characteristics and clinical relevance of circadian rhythm disorders in different metabolic organs, and the evolution of circadian-based therapeutic research.

Melatonin (N-acetyl-5-methoxytryptamine) is secreted by the pineal gland in a circadian manner as influenced by the SCN. There is also considerable evidence that melatonin, in turn, acts on the SCN directly influencing the circadian ‘clock’ mechanisms (Vriend and Reiter, 2015). In the treatment of metabolic disorders, melatonin exhibits complex yet promising effects (Zisapel, 2018). Melatonin supplementation has shown promising effects in improving sleep quality and metabolic function among the elderly. Exogenous melatonin administration alleviates non-restorative sleep, enhances circadian rhythm amplitude, and corrects circadian misalignment (Cipolla-Neto and Amaral, 2018). In patients with diabetes, sustained-release melatonin has beneficial effects in improving sleep quality and regulating sleep/wake rhythms. However, the impact of melatonin on metabolic markers varies across studies, which may be attributed to baseline metabolic status, melatonin dosage, administration timing, and individual genetic background (Arbon et al., 2015; Lauritzen et al., 2022; Martorina and Tavares, 2023; Lv et al., 2025). As an evolutionarily conserved molecule, melatonin plays a crucial role in counteracting ageing through its multifunctional actions. From the molecular mechanisms of cellular ageing to the maintenance of organ system function, melatonin has demonstrated significant therapeutic potential. Nevertheless, its complex mechanisms of action and individual variability require further investigation to achieve precise anti-ageing therapy.

## 2 Variations in circadian rhythm at the molecular level

**FIGURE 1:**
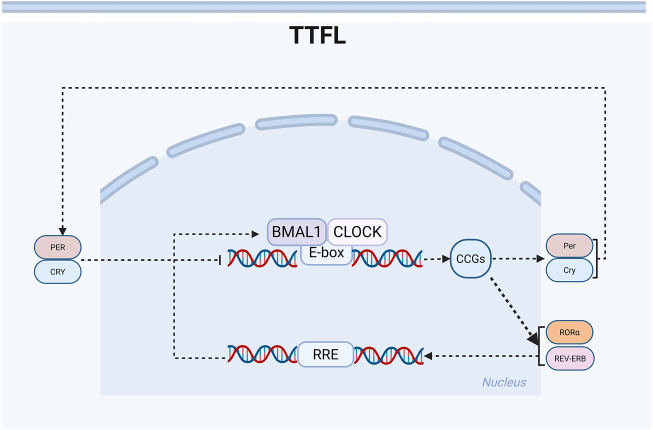
Schematic diagram of the circadian rhythm transcription-translation oscillator. Clock genes regulate the rhythm through positive and negative ‘transcription-translation feedback loops’ (TTFL). There are two main feedback loops: BMAL1: CLOCK-PER: CRY and BMAL1: CLOCK-REV-ERB/ROR. The positive regulatory elements (BMAL1, CLOCK) activate the transcription of clock control genes during the daytime phase. In contrast, the negative regulatory elements (PER, CRY, REV-ERB) inhibit transcription during the nighttime phase. The stable loop involves RORα, which activates the transcription of BMAL1, while REV-ERBα/β inhibits its transcription, thereby forming a robust 24-h oscillation.

At the molecular level, self-sustaining circadian rhythms within cells arise from a transcription-translation feedback circle. The TTFL, a transcriptional-translational feedback loop generated by the rhythmic activation of clock genes, serves as the key molecular mechanism in circadian rhythms, comprising both a primary loop and a stabilizing loop (Takahashi, 2017). Most protein-coding genes are regulated by this circuitry (Zhang et al., 2014; Mure et al., 2018). During the early circadian time (CT) phase, the transcription factors CLOCK and BMAL1 in the brain and muscle’s core loop form dimers, creating a complex that serves as a deterrent. The activation of the clock genes *Period* (*Per*) and *Cryptochrome* (*Cry*) is driven by E-box regulatory elements (Crnko et al., 2019). Post-transcriptionally, *Per* and *Cry* transcripts are translated into PER and CRY proteins in the cytoplasm, which are subsequently translocated back to the nucleus to inhibit BMAL1-CLOCK-mediated transcription, establishing a transcription-translation feedback loop. In the stabilizing loop, the nuclear receptor retinoic acid receptor-related orphan receptors (RORα) and the nuclear receptor subfamily 1 group D genes REV-ERBα (NR1D1) and REV-ERBβ (NR1D2) are activated by the CLOCK-BMAL1 heterodimer. Conversely, RORα and REV-ERBα/β sequentially stimulate and repress BMAL1 activity via the REV response element (RRE) sequence (Bugge et al., 2012; Abe et al., 2022; Hastings et al., 2023). These genes, collectively termed CLOCK-controlled genes (CCGs), exhibit rhythmic expression and drive daily oscillations in physiological functions (Sardon Puig et al., 2020). Positive regulators (BMAL1, CLOCK, NPAS2) initiate the activation of negative feedback regulators (PER, CRY, NR1D1), which in turn suppress the expression and activity of positive regulators (Sato et al., 2006) (Figure 1).

## 3 Key mechanisms regulating the circadian rhythm in the aging process

Aging is an intricate process characterized by the gradual decline of systemic and organ functions. Studies have shown that a key hallmark of aging is the attenuation of biological clock function. Aging induces multiple circadian rhythm alterations, including reduced central clock synchronization, irregular peripheral clock oscillations, and organ-specific dysfunction. This section systematically elucidates the mechanisms governing circadian rhythm disruption during aging across four domains: basic metabolism, telomere balance, epigenetic regulation, and NAD + metabolism.

### 3.1 Fundamental metabolic restructuring

Aging-induced changes in basic metabolic processes are primarily characterized by disruptions in energy metabolism, sleep-wake cycles, and metabolic circadian rhythms. Sleep serves as a fundamental biological process, influencing health and lifespan via endocrine and metabolic pathways (Pavanello et al., 2019; Liu and Reddy, 2022). Dysregulated sleep is associated with premature aging and the onset of chronic diseases (Allison et al., 2020).

Research indicates that circadian rhythm disruption induces phenotypes resembling those in 6-month-old mice with accelerated senescence (SAMP8), including cognitive impairments, metabolic dysregulation, cardiovascular abnormalities, and shortened lifespan (Akiguchi et al., 2017; Barquissau et al., 2017; Karuppagounder et al., 2017). Research on the circadian rhythm of SAMP8 mice indicates that age-related rhythm disruptions emerge as early as 7 months. Aged SAMP8 mice show reduced wheel-running activity, decreased rhythm amplitude, and increased fragmentation. These findings in SAMP8 mice are consistent with the age-related rhythm disruptions reported in healthy elderly adults, individuals with senile dementia, and other species (van Someren et al., 1996; McAuley et al., 2002; Pang et al., 2006). Compared to SAMR1 control mice, 6-month-old SAMP8 mice showed a more pronounced biphasic pattern in daily behavior (food intake and physical activity) and whole-body metabolism (energy expenditure, respiratory exchange ratio). Consistent with the delayed food intake at the end of the light phase (Bray et al., 2013), SAMP8 mice showed a phase delay (1.3–1.9 h) in the 24-h gene expression rhythms of core circadian components (BMAL1, REV-ERBα, PER2) in peripheral tissues (liver, skeletal muscle, white adipose tissue [WAT], brown adipose tissue [BAT]) (Allison et al., 2020). Conversely, restricting food intake to the dark phase enhanced metabolic oscillations and clock gene expression in peripheral tissues of both SAMP8 and SAMR1 mice.

Additionally, SAMP8 mice exhibit reduced expression of thermogenic markers (UCP1, PGC1α, DIO2) in white adipose tissue and/or brown adipose tissue, indicating decreased resistance to acute (5-h) cold exposure. SAMP8 and SAMR1 mice also show divergent responses to prolonged (1-week) ambient temperature reduction, with SAMR1 mice displaying the most robust adjustment in whole-body substrate utilization (Allison et al., 2020). Collectively, these findings reveal abnormal behavior patterns, such as *ad libitum* feeding, in prematurely aging mice, which may be linked to disruptions in peripheral circadian rhythms, metabolic processes, and thermogenesis. This further highlights SAMP8 mice as a pivotal model for advancing our understanding of age-related diseases and the mechanisms underlying circadian disruptions and aging-induced metabolic disorders.

### 3.2 Telomere abnormalities and circadian rhythm disruptions

In all vertebrate species, telomeres consist of tandem TTAGGG sequences located at the ends of linear chromosomes. Telomerase, a ribonucleoprotein complex comprising an RNA template (TERC) and a catalytic subunit (telomerase reverse transcriptase, TERT), is essential for telomere maintenance and elongation (Bernal and Tusell, 2018). Telomeric repeat sequence-containing RNA (TERRA), a non-coding RNA transcribed from telomeric regions, contributes to telomere stability. Specifically, research has shown that TERRA inhibits human telomerase activity by base-pairing with the TERC component of telomerase (Azzalin et al., 2007; Redon et al., 2010; Jones-Weinert et al., 2025).

Telomere shortening occurs rapidly during the early stages of life, coinciding with rapid growth and cellular proliferation. Reduced telomere length or accelerated telomere attrition during developmental stages is associated with shortened lifespan and health complications (Heidinger et al., 2012; Monaghan, 2014). In humans, telomerase activity declines with age, limiting the replicative lifespan of cells and affecting their function. For example, shortened telomeres in hematopoietic stem cells severely compromise their function and engraftment capacity (Fonseca Costa and Ripperger, 2015). Studies in mice have also shown that circadian rhythm disruption induces telomere shortening (Chen et al., 2014), while the circadian clock maintains homeostasis between these rhythms and telomeres, influencing telomerase activity, TERT mRNA levels, telomeric repeat-containing RNA (TERRA) expression, and telomeric heterochromatin formation (Osum and Serakinci, 2020). Circadian rhythm alterations accelerate cellular aging, with increased cellular senescence and higher prevalence of chronic diseases linked to telomere attrition (Park et al., 2019).

Zebrafish possess telomeres structurally similar to those in humans, with both species exhibiting age-related decline in telomerase functional domains, activation of telomerase by the Myc transcription factor, and regulation of telomerase activity (Carneiro et al., 2016). Over time, their telomeres gradually shorten in size. As a result, zebrafish are considered a valid and robust model for investigating the interplay among telomere attrition, aging, and disease.

Telomeres are encapsulated in heterochromatin containing H3K9me3, H4K20me3, and H3K27me3, and attach to heterochromatin protein 1 (HP1) (Schoeftner and Blasco, 2009; Galati et al., 2013). The presence of protective proteins and heterochromatin on telomeres addresses the “end-protection” problem, specifically masking, capping, and silencing chromosome ends (Perrini et al., 2004; Roig et al., 2004). Studies have revealed that the circadian rhythms of TERRA and H3K9me3 heterochromatin are lost in the brains and livers of older zebrafish (Calado and Dumitriu, 2013; Park et al., 2019). Thus, it is hypothesized that disruption of these rhythms during aging may contribute to enhanced telomere attrition in humans, given the conserved telomere biology between zebrafish and humans.

The circadian rhythm of TERRA is regulated by the BMAL1 circadian transcription factor. Research on zebrafish brains and mouse livers revealed that TERRA loses its circadian rhythm in the livers of *Bmal1* ^*−/−*^ mice, accompanied by a BMAL1-dependent H3K9me3 rhythm at telomeres that diminishes with aging (Park et al., 2019). Similarly, the absence of CLOCK and BMAL1 leads to telomere shortening through sequential inhibition of telomerase activity and the periodic expression of TERRA. Reduced telomere length promotes an increase in senescent cells with disrupted circadian rhythms. Conversely, reactivation of telomerase alleviates circadian rhythm in aging cells (Osum and Serakinci, 2020). These findings reveal a critical role for the circadian clock in maintaining telomere homeostasis by regulating TERRA and heterochromatin rhythms. This also suggests that circadian rhythm disruption during aging may contribute to telomere deterioration, providing key insights into the mechanisms linking circadian dysfunction to aging phenotypes.

### 3.3 Regulation of post-translational modifications: the influence of SIRT1 on circadian rhythms

Sirtuins, a family of class III histone deacetylases, consist of seven distinct members (SIRT1–SIRT7), each with unique functions and subcellular localizations. These proteins play a pivotal role in regulating various physiological processes, including aging. Among sirtuins, SIRT1 is involved in circadian rhythm regulation, telomere homeostasis, heterochromatin formation, metabolic pathways, DNA repair, and cellular stress responses (Asher et al., 2008; Nakahata et al., 2008; Haigis and Sinclair, 2010). Enhanced SIRT1 levels have been shown to mitigate age-related telomere deterioration in multiple tissues, highlighting a strong link between SIRT1 and telomere biology during aging, as demonstrated in both murine and human studies (Palacios et al., 2010; Amano et al., 2019).

Studies reprogramming mouse embryonic fibroblasts into pluripotent stem cells (iPSCs) have revealed that SIRT1 elongates telomeres and prevents chromosomal abnormalities after prolonged culture. A key mechanism underlying successful telomere extension during reprogramming involves SIRT1-mediated stabilization of the human cytosolic gene Myc (*c-Myc*), a regulator of mouse telomerase reverse transcriptase (mTERT) expression (De Bonis et al., 2014). Moreover, Yamashita et al. demonstrated that SIRT1 enhances transcription of human telomerase reverse transcriptase (hTERT) through a c-Myc-dependent process, thus inhibiting cellular senescence in normal human umbilical cord fibroblasts (HUC-F2) (Yamashita et al., 2012). These findings suggest that SIRT1 may modulate telomerase activity via c-Myc regulation to counteract cellular aging. Furthermore, SIRT1 might also prevent cellular senescence by alleviating telomere attrition caused by circadian rhythm disruption.

Elevated SIRT1 activity serves a dual purpose: it not only preserves telomere integrity to decelerate aging but also promotes healthy aging by regulating circadian rhythm genes (Osum and Serakinci, 2020). During mouse liver regeneration, SIRT1 ensures the precise timing and progression of cyclic gene expression to regulate the cell cycle. SIRT1 enhances the circadian rhythmicity by facilitating the deacetylation of PER2 with the mouse Clock/BMAL1/PER complex (Asher et al., 2008). Additionally, it represses PER2 expression by enhancing histone H4 deacetylation at lysine 16 (H4K16) in the PER2 promoter. Studies have shown that SIRT1 depletion results in an increased expression of PER2, thereby inducing premature aging in mice. Conversely, elevated PER2 expression inhibits SIRT1 by suppressing the CLOCK/BMAL1-driven SIRT1 transcription. This reciprocal regulatory interaction between SIRT1 and PER2 is also observed in human hepatocytes (Wang et al., 2016). As we observed in mice, the loss of SIRT1 and circadian rhythm proteins in the SCN occurs in ageing humans, which may trigger metabolic disorders and a decline in health due to the inability to re-retain them (Chang and Guarente, 2013).

SIRT1-mediated deacetylation of BMAL1 in peripheral tissues has been documented to influence circadian clock amplitude by triggering BMAL1 transcription in the SCN (Chang and Guarente, 2013). In aging normal mice, reduced SIRT1 activity and impaired BMAL1 deacetylation in the SCN lead to disrupted activity rhythms, prolonged intrinsic cycles, and defective light-entrainment. Conditional knockout of brain-specific SIRT1 expression in juvenile mice mimics these age-related circadian irregularities, while increased levels of SIRT1 expression alleviate aging impacts (Chang and Guarente, 2013). These findings suggest a potential role for SIRT1 in mitigating circadian disruptions during aging.

Conversely, it has been demonstrated that SIRT1 inhibits CLOCK-driven transcriptional activation by deacetylating H3-Lys9/Lys14 at the promoter regions of mouse clock-controlled genes (Nakahata et al., 2008). This implies that SIRT1 may exert paradoxical regulatory effects on circadian rhythms. Significantly, while most mouse and human studies highlight the beneficial effects of SIRT1 on circadian rhythms, its role in regulating these clocks during aging may involve activating CLOCK/BMAL1 formation or BMAL1 transcription, depending on its interaction network. Nonetheless, the suppressive effects of SIRT1 on circadian rhythms warrant attention and further investigation.

### 3.4 Metabolic control: the oscillatory role of NAD^+^

Nicotinamide adenine dinucleotide (NAD^+^) plays a pivotal role in controlling biosynthesis and cellular metabolism (Imai and Guarente, 2014; Garten et al., 2015). The enzyme nicotinamide phosphoribosyl transferase (NAMPT) is central to maintaining cellular NAD^+^ levels (Revollo et al., 2004), with the *NAMPT* gene expression regulated by core circadian mechanisms via sirtuins. SIRT1, an NAD^+^-dependent deacetylase, is integral to circadian rhythm regulation. Studies have shown that a direct link between cellular energy metabolism and biological oscillations. SIRT1 interacts with the BMAL1/CLOCK heterodimer (a clock-activating complex) to modulate NAMPT transcription (Nakahata et al., 2009; Ramsey et al., 2009), highlighting a critical enzymatic feedback loop where the SIRT1/BMAL1/CLOCK axis controls metabolic processes through NAD^+^ activity. In this context, SIRT1 resides at the interface of the NAMPT transcriptional regulatory loop and multiple biological functions via NAD^+^, serving as a metabolic oscillator (Imai, 2010).

As a crucial regulatory hub, the hypothalamus coordinates metabolic processes, circadian rhythms, and age-related physiological homeostasis (Saper et al., 2005). NAD^+^ oscillations are essential for hypothalamic function, and their disruption contributes to aging-associated circadian and metabolic disorders (Tokizane and Imai, 2021). With aging, systemic NAD^+^ depletion impairs hypothalamic neuronal function, leading to age-related metabolic pathologies such as obesity and chronic diseases (Imai and Guarente, 2014; Roh et al., 2018). Studies indicate that supplementation with the NAD^+^ precursor nicotinamide riboside (NR) robustly modulates age-diminished metabolic and stress-response pathways while inhibiting the clock inhibitor PER2. NR enhances genome-wide BMAL1’s chromatin binding by regulating PER2(K680) deacetylation, implying NAD^+^ may trigger PER2 deacetylation via SIRT1 to control circadian rhythms. Notably, PER2 deacetylation influences the phosphorylation at the CK1 site on PER2, a mechanism linked to human hereditary late sleep phase syndrome (Toh et al., 2001), further validating its role in circadian rhythms.

Following NAD^+^ supplementation, age-related declines in BMAL1 chromatin binding, transcriptional oscillations, mitochondrial respiration, and nocturnal activity in mice were restored to youthful levels (Levine et al., 2020). These findings indicate that NAD^+^ influences aging-associated metabolic and circadian dysfunction by regulating clock gene expression. A potential therapeutic strategy involves enhancing SIRT1 activity via NAD^+^ to alleviate age-related behavioral and metabolic decline (Ondracek et al., 2017). At the cellular level, targeting PER2 K680 acetylation to reset circadian rhythms may offer a therapeutic avenue for disorders such as neurodegeneration-linked sleep loss and shift work disorders.

## 4 Impact of circadian rhythm disorders on metabolic organs and their molecular mechanisms

With aging, the decline of circadian rhythms can lead to functional abnormalities in multiple metabolic organs. This section systematically explores how circadian disruptions affect metabolic organs and their underlying molecular mechanisms.

### 4.1 Circadian rhythm disruption and pancreatic islets

Pancreatic islets, as critical endocrine organs, regulate glucose metabolism through intricate circadian oscillations. The insulin secretion by pancreatic β-cells, glucagon release by α-cells, and glucose sensitivity each exhibit distinct circadian patterns. Research indicates significantly reduced mRNA levels of core clock genes (e.g., PER3, PER2, and CRY2) in pancreatic islets from type 2 diabetes mellitus (T2D) patients, suggesting dysregulation of pancreatic islet cell circadian rhythms (Stamenkovic et al., 2012). Inhibition of CLOCK expression in human islet cells leads to increased BMAL1 and CRY1 levels and decreased REV-ERB and PER3 expression. Conversely, circadian clock disruption impairs insulin secretion during both acute and chronic glucose stimulation, while basal insulin release from synchronized human islet cells *in vitro* disrupts circadian rhythms. The mRNA expression of BMAL1, PER1, and PER3 in white blood cells from patients with T2D was negatively correlated with hemoglobin A1c (HbA1c) levels. In pancreatic islets of T2D patients or healthy controls, the mRNA expression of PER2, PER3, and CRY2 was positively correlated with islet insulin content and plasma HbA1c levels (Stamenkovic et al., 2012). These findings indicate that the expression of molecular clock genes is associated with T2D and insulin resistance.

RNA sequencing study showed significant changes in the expression levels of 352 transcripts due to disruptions in biological clock regulation. The modifications primarily involved two categories of elements: 1) key regulators associated with the insulin release pathway, including GNAQ (G protein-coupled receptor signaling), ATP1A1 (Na^+^/K^+^ ATPase), ATP5G2 (mitochondrial ATP synthesis), and KCNJ11 (ATP-sensitive potassium channel); and 2) genetic sequences related to insulin granule biogenesis and secretion, such as VAMP3 (vesicle-associated membrane protein 3), STX6 (synaptic fusion protein 6), and SLC30A8 (zinc transporter 8) (Stamenkovic et al., 2012; Dibner and Schibler, 2015; Saini et al., 2016).

In circadian mutant mice, where the oscillatory phase of islet genes involved in growth, glucose metabolism, and insulin signaling is delayed, both *Clock* and *Bmal1* mutants exhibit age-progressive impairments in glucose tolerance, reduced insulin secretion, and defects in islet size and proliferation that worsen with age (Bunger et al., 2000; Turek et al., 2005; Marcheva et al., 2010). Disruption of the circadian clock leads to widespread alterations in the expression of islet genes implicated in cell growth, survival, and synaptic vesicle biogenesis (Marcheva et al., 2010). Acting as anti-aging regulators, CLOCK and BMAL1 are critical for maintaining glucose metabolism and insulin function during aging, along with their interacting pathways.

Significantly, circadian-disrupted alterations in mammalian glucolipid metabolism exhibit profound sex differences. Research on juvenile (8-week-old) male and female mice demonstrates that restricting food intake to a 6-h window during the night/active period impairs glucose tolerance, potentially attributable to circadian disruption induced by this feeding regimen. Although both sexes showed comparable changes in feeding behavior, body structure, and various metabolic parameters, significant sex-specific differences were observed in the magnitude and pattern of these changes. Female rodents exhibit enhanced fat and triglyceride storage capacity, as evidenced by lower fasting insulin levels, and exhibit more significant shifts in energy utilization, particularly in the mobilization of alternative energy sources (e.g., fat via β-oxidation) (Freire et al., 2020). These findings highlight the inherent biological and metabolic disparities between male and female mice and underscore the critical need to include both sexes in scientific research.

These findings provide critical insights into the circadian regulatory mechanisms of pancreatic islets and their age-related dynamics, offering a theoretical foundation for the development of innovative therapeutic strategies. For example, optimizing drug administration schedules and designing gender-tailored treatment plans. Furthermore, the results emphasize the requirement to prioritize gender disparities in future research to devise more precise therapeutic strategies.

### 4.2 Disruption in liver metabolism and circadian rhythms

As a vital metabolic organ maintaining metabolic homeostasis (He et al., 2023a), the liver’s function is tightly regulated by circadian rhythms. Studies have shown that approximately 10%–15% of liver genes exhibit circadian rhythmicity, playing critical roles in essential physiological processes such as glucolipid metabolism, bile acid synthesis, and drug metabolism (Nohara et al., 2020). Age-related changes disrupt this regulatory network (Panda et al., 2002; Hood and Amir, 2017; Guan and Lazar, 2021). Research has revealed that in aged mice, 44.8% of genes with rhythmic expression in young mouse liver tissue exhibited rhythmic abnormalities, predominantly affecting glycerol and sterol metabolism pathways (Sato et al., 2017). Significantly, the lack of certain circadian genes (e.g., Bmal1 and Per1/2) accelerates liver aging (Froy, 2011). Consequently, during liver aging, the interplay between lipid metabolic dysfunction and circadian rhythm disruption may escalate, although the mechanisms underlying their synchronization remain unclear.

Research indicates that the transcription factor early growth response-1 (Egr-1), a member of the immediate early gene family, binds to highly conserved GC-rich promoter sequences to regulate the expression of numerous target genes (Silverman and Collins, 1999). Egr-1 can be activated by various stimuli, including the hepatic clock circuit (Chavrier et al., 1988; Tao et al., 2015). Recent research has shown that Egr-1 is critical for regulating hepatic circadian rhythms. In young mice, Egr-1 exhibits rhythmic expression in the liver, modulating the circadian rhythmicity of key clock genes, particularly controlling the transcriptional activity of the biorhythm gene *Per1*. Conversely, the rhythmic activity of the BMAL1/CLOCK heterodimer also regulates Egr-1 expression (Tao et al., 2015). Egr-1 gene expression is markedly reduced in multiple aging cell lines and in the livers of 21-month-old mice (Bochkis et al., 2014; Penner et al., 2016). Collectively, these findings imply that Egr-1 may play a role in regulating age-related circadian rhythms and hepatic metabolism.

With aging, the peak expression time (ZT) of the transcription factor Egr-1 advances. In the liver, Egr-1 rapidly maintains the normal rhythms of the peripheral clock (Tao et al., 2015). Upon activation by the central circadian signal, Egr-1 binds to the *Per1* gene promoter to initiate its transcription, thereby repressing *Per2* and *Rev-erbs*. Suppression of Rev-Erbs leads to strong oscillations in BMAL1, which subsequently bind to and activate Egr-1. The Egr-1/Per1/BMAL1/Egr-1 feedback loop may help synchronize the liver clock with the central clock, thereby preserving the amplitude of circadian rhythms (Tao et al., 2015).

**FIGURE 2:**
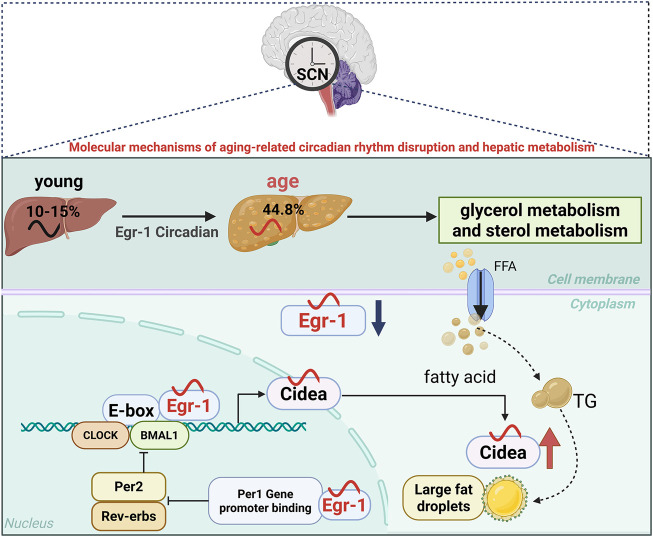
Schematic illustration of age-associated circadian rhythms in liver tissue. The figure illustrates the gradual deterioration of the liver’s circadian rhythm function with aging, as follows: In a young liver, a robust Egr-1/Per1/BMAL1/Egr-1 feedback loop maintains normal lipid metabolism and clock gene expression; aged livers exhibit reduced Egr-1 expression, disrupted circadian gene oscillations, and impaired Egr-1/Cidea interactions, which lead to lipid droplet accumulation. During aging, the peak expression time (ZT) of the transcription factor Egr-1 shifts earlier with increasing age. This demonstrates how aging disrupts the coordination between metabolic and circadian pathways. Blue arrows indicate age-related decreases in gene expression amplitude, while red arrows indicate increased lipid droplet accumulation.

Subsequent research revealed that Egr-1 forms a complex with the circadian transcription factors BMAL1/CLOCK to regulate the transcription of lipid metabolism-related genes, such as cell death-induced DFFA-like effector A (*Cidea*). The coordinated interaction between Egr-1 and Cidea controls the formation of substantial lipid droplets in a BMAL1/CLOCK-dependent manner (Wu et al., 2023). However, aging disrupts the Egr-1/Cidea interaction, leading to excessive lipid droplet accumulation and subsequent age-related hepatic metabolic dysfunction (Wu et al., 2023). Therefore, the decline of Egr-1 expression with aging and the disruption of its circadian rhythm may represent a key mechanism underlying age-related hepatic metabolic decline. Egr-1 appears to be a critical regulator of the crosstalk between hepatic circadian rhythms and age-related lipid metabolism (Figure 2).

### 4.3 Disruption of circadian rhythms in adipose tissue

Adipose tissue serves as both an energy storage organ and a key endocrine organ, whose functions are tightly regulated by circadian rhythms. Research indicates that approximately one-quarter of genes in adipose tissue display circadian rhythmicity, predominantly influencing lipid metabolism, glucose transport, and inflammation (Adamovich et al., 2015). Significantly, white adipose tissue (WAT) and brown adipose tissue (BAT) display distinct rhythmic characteristics. WAT is primarily responsible for energy storage, with its circadian rhythm closely associated with feeding behavior (Kwon and Park, 2024), while BAT mainly regulates energy expenditure through thermogenesis, and its rhythm is controlled by the sympathetic nervous system. With aging, the circadian rhythmicity of adipose tissue gradually declines, leading to metabolic disorders and insulin resistance (He et al., 2023b; Yang et al., 2025).

The nuclear receptor peroxisome proliferator-activated receptor γ (PPARγ), a critical mediator linking metabolism and circadian rhythms, plays a significant role in regulating lipid synthesis and utilization, glucose uptake, hormonal responses, and adipokine production in adipose tissue (Ahmadian et al., 2013). It also directly controls the expression of circadian rhythm-related genes. PPARγ has been shown to reprogram hepatic circadian rhythms under overnutrition conditions (Eckel-Mahan et al., 2013), and mediate the BMAL1 downregulation in the adipose tissue of obese mice and humans (Wang S. et al., 2022).

Recent research has shown that PPARγ activity is regulated by complex post-translational modifications (PTMs), with acetylation being particularly crucial (Brunmeir and Xu, 2018). Specifically, deacetylation of PPARγ at Lys268 and Lys293 by the deacetylase SIRT1 facilitates brown adipocyte trans differentiation of WAT (Qiang et al., 2012), enhances energy expenditure, and protects against obesity. By generating a constitutively deacetylated PPARγ mimic mutant (K268R/K293R, also known as 2 KR), researchers found that this modification selectively regulates downstream genes and dissociates metabolic benefits from adverse effects such as bone loss (Kraakman et al., 2018; Liu et al., 2020; Aaron et al., 2021). These findings demonstrated that PPARγ acetylation in adipose tissue orchestrates daily metabolic oscillations.

In young, healthy mice, PPARγ acetylation levels show a robust circadian rhythm, peaking at Zeitgeber time 0 (ZT0) and reaching a nadir at ZT18. However, this rhythmic pattern is disrupted in the context of aging and obesity (He et al., 2023a). A comprehensive study revealed that adipocyte-specific acetylation-mimicking PPARγ K293Q (aKQ) impairs adipose tissue plasticity by promoting degradation of the core circadian component BMAL1 (Wang S. et al., 2022). Studies on aKQ and reciprocal 2 KR mutant mice revealed notable changes in the circadian patterns of glucose tolerance and insulin sensitivity (He et al., 2023a). Collectively, PPARγ acetylation appears to be a critical mode linking adipose tissue adaptability to metabolic cycles, whereby aging and obesity disrupt lipid metabolism. PPARγ acetylation also regulates circadian rhythm disruption and age/obesity-related glucolipid metabolic abnormalities.

**FIGURE 3:**
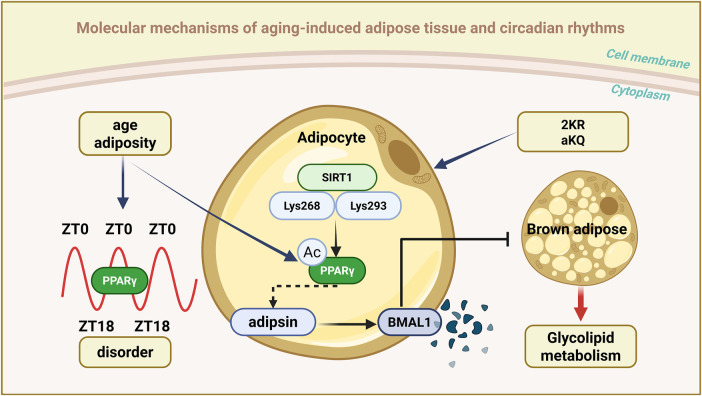
Schematic representation of aging-associated circadian rhythms in adipose tissue. The figure illustrates the role of PPARγ acetylation in coordinating circadian metabolic rhythms: In young adipose tissue, PPARγ exhibits a robust circadian acetylation pattern (peaking at ZT0 and terminating at ZT18), maintaining appropriate stability of BMAL1 and metabolic flexibility; During ageing and obesity, this rhythmic acetylation is disrupted, leading to adipsin-mediated BMAL1 degradation and metabolic dysfunction. This pathway demonstrates how SIRT1-mediated deacetylation of PPARγ (particularly at K268/K293) promotes healthy metabolic oscillations, while constitutive acetylation (simulated by the K293Q mutation) impairs circadian clock function and adipose tissue plasticity.

The specific regulation of downstream targets via PPARγ deacetylation, particularly adipsin—a key adipokine associated with obesity, aging, and type 2 diabetes—is of significant interest (Kraakman et al., 2018; Aaron et al., 2021). Researchers have identified complement factor D (also known as adipsin) as a novel circadian regulator modulated by PPARγ acetylation. Adipsin is known to specifically facilitate BMAL1 protein degradation, induce BMAL1 instability, and regulate metabolic rhythms (He et al., 2023a). This regulatory mechanism is adipocyte-specific; however, despite the high co-expression of adipsin and PPARγ in these cells, a direct interaction between them has not been detected (He et al., 2023a). This implies the involvement of additional intermediate molecules in adipsin-mediated regulation of BMAL1. Specifically, adipsin-induced BMAL1 degradation may require PPARγ to initiate certain downstream targets, thereby facilitating BMAL1 degradation by adipsin. Although the circadian regulation of PPARγ in metabolism has been extensively studied (Guan and Lazar, 2021), adipsin-mediated BMAL1 instability underscores a potentially novel pathway for circadian clock dysregulation in aging and obesity (Chen et al., 2016; Wang S. et al., 2022) (Figure 3).

### 4.4 Disorders in skeletal muscle metabolism and circadian rhythms

Skeletal muscle, which accounts for 40%–50% of total human body weight and serves as the primary metabolic organ, plays a dual role in locomotion and glycolipid metabolism. Research indicates over 2,300 genes in skeletal muscle exhibit notable circadian rhythmicity, predominantly regulating glucose transport, lipid oxidation, and mitochondrial function (Harfmann et al., 2015; Martin et al., 2023). With aging, the circadian rhythmicity of skeletal muscle declines, resulting in metabolic dysfunction and reduced muscular strength.

The circadian clock is increasingly recognized as a key regulator of skeletal muscle physiology (Harfmann et al., 2015; Schroder et al., 2015). Skeletal muscle serves as the primary site of glucose disposal, and disruption of the muscle clock via B*mal1* knockout (KO) leads to impaired glucose homeostasis (Dyar et al., 2014). Disruption of the muscle clock attenuates the whole-body glucose utilization, particularly during the early stages of glycolysis, affecting the switch from anabolic to catabolic glucose fates, as demonstrated in skeletal muscle-specific *Bmal1* knockout (mKO) mouse model (Chaikin et al., 2025). Compared to wild-type mice, mKO mice exhibit elevated fasting glucose levels, reduced insulin sensitivity, and approximately 37% decreased skeletal muscle glucose uptake (Dyar et al., 2014). Mechanistic studies have shown that Bmal1 regulates glucose metabolism in skeletal muscles by directly controlling the expression and trafficking of glucose transporter 4 (GLUT4). In mKO mice, insulin-induced GLUT4 translocation efficiency is reduced, GLUT4 transcript and protein levels are decreased, and protein levels drop by 45% (Schiaffino et al., 2016).

Research using systemic ClockΔ19 mutant and *Bmal1* KO mouse models indicate a strong link between core circadian clock components and skeletal muscle aging. These mutant mice displayed prominent features such as misaligned muscle fiber organization, reduced cross-sectional size, and impaired contractile function, indicating the essential role of the circadian clock in maintaining muscle architecture and physiological function (Andrews et al., 2010). Skeletal muscle-specific *Bmal1* KO also affects exercise capacity, with a 48% reduction in the number of exercise-responsive genes in *Bmal1* KO muscle, with 517 genes differentially expressed (250 upregulated and 267 downregulated) (Viggars et al., 2024). Activation of immediate-early genes (e.g., *Nr4a3* and *Ppargc1a*) induced by acute exercise requires the presence of Bmal1 in skeletal muscle (Viggars et al., 2024). Concurrently, trained mice skeletal muscle specific BMAL1 deficiency exhibited inflammation in the liver, adipose tissue, and lungs (Viggars et al., 2024).

Moreover, BMAL1 in skeletal muscles is essential for maintaining post-exercise metabolic balance of pyruvate and crucial TCA cycle intermediates (Viggars et al., 2024). The circadian clock’s transcription-translation feedback loop tightly regulates cellular energy metabolism hubs, including mitochondrial biosynthesis, fusion/fission dynamics, and quality control processes such as mitophagy (Manella and Asher, 2016; de Goede et al., 2018; Figueroa-Toledo et al., 2024). Regarding mitochondrial fusion-fission balance, studies in cells, mice, and humans indicate that reduced BMAL1/CLOCK protein levels correlate with enhanced mitochondrial-sarcoplasmic reticulum interactions, decreased OPA1 and DRP1 protein levels (key regulators of mitochondrial fusion and fission), diminished activation of protein synthesis signaling pathways (like mTOR, AKT, and P70S6K), and compromised skeletal muscle integrity. These factors are closely associated with the aging process (Tezze et al., 2017; Favaro et al., 2019; Castro-Sepulveda et al., 2023).

Studies in humans have shown that rhythmic mitochondrial metabolic dysfunction may mediate the disrupted rhythmic cellular metabolism and molecular clock mechanisms in primary muscle tubules of patients with T2D. The circadian rhythmicity of skeletal muscle mitochondria is more likely derived from mitochondrial membrane dynamic processes, which are regulated by rhythmic proteins such as OPA1 and FIS1. Mitochondrial dysfunction in peripheral tissues is associated with insulin resistance and the pathogenesis of T2D, accompanied by increased ROS production, reduced oxidative capacity, and decreased metabolic flexibility (Gabriel et al., 2021). Thus, disruption of the daily rhythmicity of mitochondrial activity may contribute to skeletal muscle insulin resistance in T2D. Additionally, mitochondrial membrane dynamics exhibit circadian rhythmic behavior in several peripheral tissues (de Goede et al., 2018); however, the transcriptional control of these pathways is tissue-specific (Weaver et al., 2014; Gabriel et al., 2021). For example, Drp1, Mfn1/2, and Opa1 are not direct targets of the hepatic clock mechanism (Jacobi et al., 2015), but Mfn1 and Opa1 are reduced in the myocardium of Bmal1^−/−^ mice (Kohsaka et al., 2014).

Notably, the synthesis of cardiolipin (CL), a key lipid in the mitochondrial inner membrane, is regulated by circadian rhythms. Research indicates rhythmic gene activity in C2C12 cells for CL biosynthesis (Taz and Ptpmt1), and disruptions in the *Ror* gene, encoding the ROR nuclear receptor in the circadian oscillator’s secondary loop, result in altered or reduced expression of Taz and Ptpmt1. This demonstrates that CL levels oscillate across the circadian cycle in C2C12 myotubes (Nohara et al., 2020). Compared to young mice, aged mice exhibited elevated CL levels in skeletal muscle. Nobiletin, a natural ROR agonist, partially restored CL synthesis gene expression in aged muscles via dietary intervention (Nohara et al., 2020). These findings underscore the circadian-dependent cyclical production of CL in skeletal muscle, which is regulated by RORs and modulated by dietary factors and age.

The maintenance of skeletal muscle regenerative capacity depends on the proper function of satellite cells (MuSC). Myo-microRNAs (MiRs) are skeletal muscle-specific miRNAs that play critical roles in regulating the expression of various proteins essential for myogenesis and the proliferation, differentiation, and maintenance of MuSC (Horak et al., 2016; Mok et al., 2017). Recent research has shown that the Musashi 2 (Msi2) protein impairs muscle regeneration during aging by disrupting clock gene function, in part through regulating microRNA biogenesis. Msi2 is highly expressed in mature muscle cells and promotes MuSC differentiation. Additionally, Msi2 modulates MiR biogenesis during myogenesis and MuSC aging (Siddall et al., 2006; Ito et al., 2010; de Andrés-Aguayo et al., 2011).

Mechanismic research reveals that Msi2 interacts with the RNA-binding protein human antigen R (HuR) in a dose-dependent manner to suppress MiR7a-1 formation. Additionally, Msi2 and HuR enhance the activity of cryptochrome ci rcadian regulator 2 (Cry2), a core component of the circadian oscillator Cry2 complex that controls MuSC differentiation by repressing MiR7a-1 biogenesis (Yang et al., 2022). In aged muscles, both Msi2 expression and Cry2 protein levels are reduced (Yang et al., 2022), indicating that the disruption of the Msi2-driven post-transcriptional circadian regulatory cascade may underlie the decline in muscle regenerative ability in aged skeletal muscle. Collectively, the age-related loss of dosage balance between Msi2 and HuR disrupts the microRNA biogenesis regulatory cascade, potentially resulting in impaired MuSC differentiation in aged mice—a process with relevance to human aging.

**FIGURE 4:**
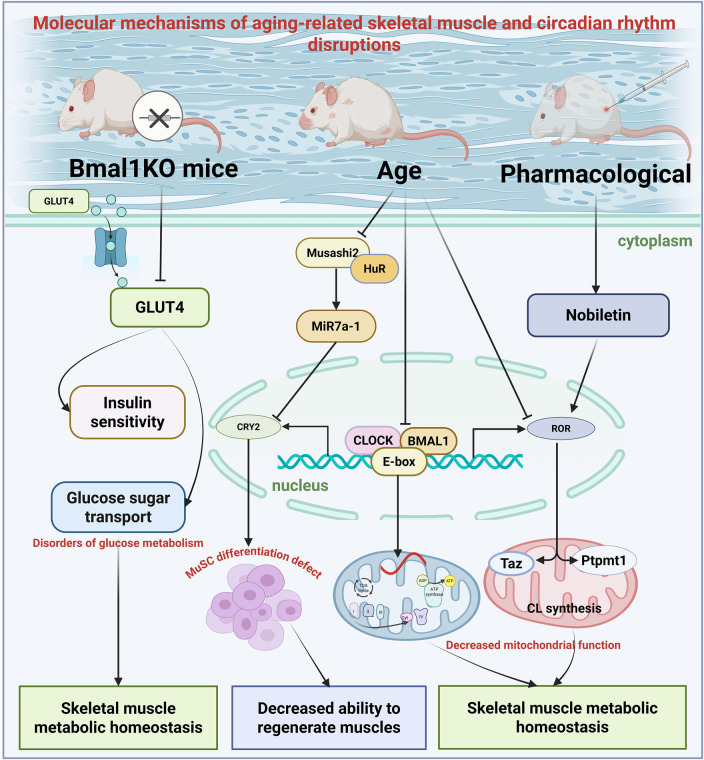
Schematic illustrations of the circadian rhythms in aging skeletal muscle tissue. This figure illustrates the complex interactions between circadian clock components and muscle function during the aging process: Young muscles exhibit coordinated regulation of glucose metabolism (via GLUT4), mitochondrial dynamics (OPA1/DRP1 balance), and satellite cell function (MuSC) by BMAL1/CLOCK; Aged muscles exhibit disrupted circadian rhythm control, with reduced BMAL1 expression leading to impaired glucose uptake, mitochondrial dysfunction, and reduced regenerative capacity. The Msi2/HuR/Cry2 pathway illustrates how age-related changes in RNA-binding proteins affect microRNA biogenesis and circadian clock function. Additionally, CL synthesis rhythms are regulated by ROR nuclear receptors and are influenced by the ROR agonist Nobiletin and dietary interventions.

Based on the understanding of these mechanisms, approaches like time-restricted feeding and optimizing exercise timing have shown promise in improving skeletal muscle function in older adults. For example, restricting daily feeding to 8–10 h markedly enhanced mitochondrial respiration by 35% and muscular strength by 28% in aged mice (Sato et al., 2019). Morning workouts (ZT2–4) improve skeletal muscle metabolism and increase glucose uptake efficiency by approximately 25% (Ezagouri et al., 2019). Additionally, pharmacological interventions targeting the ROR signaling pathway have demonstrated therapeutic potential, with the ROR agonist Nobiletin enhancing mitochondrial function in aged muscles by restoring CL synthesis (Nohara et al., 2020). These findings deepen our understanding of how skeletal muscles regulate circadian rhythms and provide innovative strategies for preventing and treating age-related muscle dysfunction (Figure 4).

### 4.5 Disruption in bone structure and circadian rhythms

Bone tissue functions as both the body’s structural support and a metabolically active organ with circadian rhythmicity. During aging, disruptions in bone tissue circadian rhythms may lead to abnormal bone metabolism, affecting bone health. The circadian system rigorously controls the differentiation and function of osteoblasts, cells in bone tissue. Research revealed that Bmal1-deficient osteoblasts exhibit specific phenotypes: increased osteogenic marker expression, reduced alkaline phosphatase activity, impaired mineralization, enhanced apoptosis, and intensified inflammatory responses (Li et al., 2023). BMAL1 expression decreases in human osteoarthritis and aged mouse cartilage (Charlesworth et al., 2006). BMAL1 suppression in chondrocytes promotes cell proliferation and upregulates MMP13 expression (Cragle and MacNicol, 2014). Progressive deterioration of knee cartilage occurs in BMAL1-deficient mice (Charlesworth et al., 2006). Furthermore, aging and ongoing inflammation disrupt autonomous circadian patterns in mouse cartilage (Murphy et al., 2016). These findings suggest that age-related circadian rhythm disruptions contribute significantly to the development of skeletal disorders.

Comprehensive mechanistic research has shown that BMAL1 regulates osteogenic differentiation via modulation of the mTOR/GSK3β/β-catenin signaling pathway. In *Bmal1*-deficient cells, increased mTOR activity suppressed the GSK3β/β-catenin pathway, leading to reduced β-catenin levels and phosphorylation of GSK-3β at serine 9, which in turn resulted in defects in osteoblastic differentiation. Research indicates that Bmal1 controls osteoblast differentiation and inflammatory responses in an mTOR/GSK3β/β-catenin-dependent manner, potentially influencing bone mineralization and remodeling. Significantly, treatment with TDZD-8 (a GSK3β inhibitor) or rapamycin (an mTOR inhibitor) partially reversed BMAL1 loss-induced suppression of β-catenin expression and GSK-3β phosphorylation (Li et al., 2023), thereby alleviating osteogenic differentiation defects and indicating the therapeutic potential of these agents for circadian-related bone metabolic disorders.

With advancing age, the incidence of heterotopic ossification in tendons and ligaments increases significantly (Liang et al., 2022), which is closely associated with circadian rhythm disruption. Temporal analysis showed that *Bmal1*-deficient mice developed age-dependent progressive heterotopic ossification starting at 6 weeks of age, predominantly impacting the Achilles tendon and posterior longitudinal ligament. Achilles tendon ossification was a gradual decline in ankle joint motor abilities, along with a notable increase in cartilage formation within the posterior longitudinal ligament and Achilles tendon.

Molecular mechanistic investigations showed that BMAL1-deficient ligament-derived fibroblasts exhibited upregulated osteogenic and chondrogenic markers, activation of the TGF-β/BMP signaling pathway, and enhanced TGF-β1-induced osteogenic differentiation capacity. Furthermore, BMAL1-deficient mouse embryonic fibroblasts exhibit increased osteogenic differentiation potential upon TGF-β/BMP signaling activation (Liang et al., 2022). These findings indicate that BMAL1 suppresses heterotopic ossification in tendons and ligaments by inhibiting TGF-β/BMP signaling-mediated endochondral ossification, revealing a novel circadian rhythm-dependent regulatory mechanism for age-related tendon and ligament heterotopic ossification.

Biological clocks regulate tissue function by controlling tissue-specific gene expression programs. In articular cartilage, circadian rhythm governs the expression of 615 genes, accounting for 3.9% of all transcripts in cartilage tissue (Gerstberger et al., 2014). Most peripheral tissues and cultured cells, including cartilage and chondrocytes, possess autonomic clocks (Siddall et al., 2006; Gerstberger et al., 2014; Murphy et al., 2016; Kharas and Lengner, 2017). Normal human chondrocytes show robust circadian rhythms of NR1D1 and BMAL1. In osteoarthritis (OA), NR1D1 and BMAL1 mRNA and protein levels are significantly reduced compared to healthy cartilage (Baghdadi and Tajbakhsh, 2018). RNA sequencing of chondrocytes transfected with NR1D1 or BMAL1 siRNA identified 330 and 68 differentially expressed genes, with major impacts on the TGF-β signaling pathway (Baghdadi and Tajbakhsh, 2018).

Dysregulated expression of circadian genes in chondrocytes contributes to OA pathogenesis. Recent research indicates a significant role of CRY2 in maintaining cartilage homeostasis. Earlier RNA-sequencing analyses revealed imbalanced expression of the core circadian clock gene CRY2 in human OA cartilage (Zearfoss et al., 2014). Notably, CRY2 staining, and mRNA expression are significantly reduced in human OA cartilage. In OA chondrocytes, both CRY1 and CRY2 levels are diminished, accompanied by attenuated circadian rhythm amplitude. Additionally, Cry2 expression is downregulated in the aging-related OA mouse model (Bekki et al., 2020). Mechanistically, Cry2 KO exacerbates histopathological changes in the cartilage, subchondral bone, and synovium of OA mice.

**FIGURE 5:**
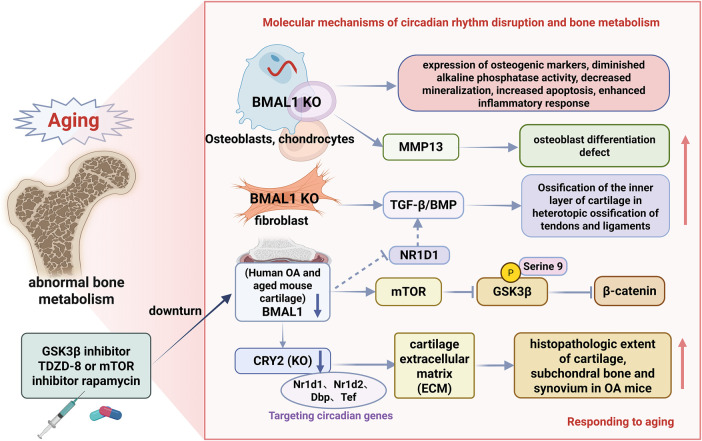
Schematic representation of aging-associated circadian rhythms in bone tissues. This figure illustrates the multifaceted roles of BMAL1 in bone metabolism: In a normal bone homeostasis state, BMAL1 mediates the regulation of osteoblast differentiation through the mTOR/GSK3β/β-catenin pathway, prevents ectopic ossification by inhibiting TGF-β/BMP signaling, and maintains cartilage integrity through CRY2-dependent extracellular matrix homeostasis; BMAL1 deficiency leads to age-related bone pathology by promoting mTOR activity, inhibiting GSK3β/β-catenin signaling, and activating the TGFβ/BMP signaling pathway, resulting in abnormal osteoblast differentiation, osteogenesis disorders, increased ectopic ossification of tendons and ligaments, and cartilage degeneration associated with osteoarthritis. The figures illustrate therapeutic interventions (TDZD-8, rapamycin) and their molecular targets, demonstrating potential treatment strategies for circadian rhythm-related bone diseases.

RNA sequencing of knee cartilage from wild-type and Cry2 KO mice identified 53 differentially expressed genes, including the known circadian genes *Nr1d1*, *Nr1d2*, *Dbp*, and *Tef* as direct targets of Cry2. Pathway analyses revealed circadian rhythm disruption and extracellular matrix (ECM) remodeling abnormalities in Cry2-deficient mice (Bekki et al., 2020). Collectively, CRY2-mediated circadian rhythms are essential for maintaining the cartilage ECM homeostasis. This autonomous circadian gene network is dysregulated in both normal aging and OA chondrocytes. Targeting CRY2 may normalize aberrant gene expression profiles and mitigate OA severity (Figure 5).

## 5 Melatonin-mediated regulation of circadian rhythms associated with aging

Melatonin, as the primary hormone secreted by the pineal gland, serves as a key messenger molecule linking the central circadian rhythm clock to peripheral metabolic processes. With advancing age, melatonin secretion exhibits a marked age-related decline, a change closely associated with the overall aging of the circadian rhythm system and increased risks of various metabolic disorders. This chapter will comprehensively discuss the important role of melatonin in the aging process and its clinical application prospects from aspects such as age-related changes in melatonin, receptor mechanisms, metabolic regulatory effects, and anti-aging mechanisms.

### 5.1 Melatonin-mediated aging-related circadian rhythm regulation mechanisms

With age, melatonin secretion in the human body shows a significant decline, a process closely related to the deterioration of the stability of the circadian rhythm system. Abnormal circadian rhythms and poor sleep quality are associated with increased risks of cardiovascular, metabolic, and cognitive diseases, deteriorated quality of life, and elevated mortality rates (Zisapel, 2018; Fernández-Martínez et al., 2023). Age-related decline in pineal gland function is the primary cause of melatonin deficiency. Melatonin production decreases significantly with age, potentially leading to circadian rhythm disorders and disruption of clock genes in various tissues. The reduction in melatonin levels in age-related tissues appears to be associated with mitochondrial dysfunction occurring during this process (Fernández-Martínez et al., 2023). Melatonin supplementation can maintain more normal mitochondrial physiology in aging neurons. Melatonin can mitigate Alzheimer’s disease (AD) pathology and improve cognition by improving mitochondrial function. This suggests a vicious cycle between melatonin synthesis levels and aging: melatonin synthesis decreases with age, and simultaneously, the aging process worsens due to melatonin deficiency (Zhao et al., 2019; Chen et al., 2021).

### 5.2 Mechanism of melatonin regulation of islet function

Melatonin exhibits complex time-dependent characteristics in regulating pancreatic cell function. A study showed that patients with prediabetes and insulin resistance exhibit lower excretion levels of melatonin metabolites (6-sulfoxy melatonin) in urine (Reutrakul et al., 2018). It is well-described that pancreatic alpha and beta-cells express melatonin receptors MT1 and MT2, and evidence shows that melatonin action on the pancreatic melatonin receptors modulates insulin secretion and glucagon release in a diurnal fashion (Speksnijder et al., 2024). However, the effect of melatonin on insulin secretion is time sensitive. When elevated melatonin levels coincide with mealtimes, particularly during nighttime eating or when melatonin levels rise during the day, high melatonin levels may inhibit insulin release and/or insulin sensitivity, leading to impaired glucose tolerance (Amaral et al., 2019; Martorina and Tavares, 2023; Speksnijder et al., 2024).

Many studies have also investigated the relationship between genetic polymorphisms of the melatonin MTNR1B receptor gene and increased susceptibility to type 2 diabetes mellitus and circadian rhythm disorders (Zhang et al., 2020; Zhu et al., 2023). Genetic variations in the MTNR1B gene further complicate the metabolic effects of melatonin. Individuals carrying the rs10830963 risk variant of the MTNR1B gene exhibit enhanced MT2 receptor function, making cells more sensitive to the effects of melatonin. These gene variant carriers experience enhanced insulin secretion inhibition by melatonin in response to glucose stimulation when endogenous melatonin levels rise at night, thereby increasing their risk of postprandial hyperglycemia (Zhu et al., 2023).

Human studies have shown that reduced melatonin secretion increases insulin resistance and the incidence of type 2 diabetes (McMullan et al., 2013a; McMullan et al., 2013b). Additionally, a clinical trial involving patients with type 2 diabetes and insomnia demonstrated that long-term administration of sustained-release melatonin improved glycemic control and suppressed hypertriglyceridemia and hyperinsulinemia (Garfinkel et al., 2011). Conversely, two placebo-controlled studies indicated that acute melatonin supplementation in the morning and evening had harmful effects on glucose tolerance and insulin sensitivity in both young and elderly women (Rubio-Sastre et al., 2014). Although the above studies provide evidence of melatonin’s beneficial effects on circadian rhythms and blood glucose control, recent research has only partially elucidated the causal relationship between melatonin signaling and T2D risk. Genetic effects may also be crucial in the development of T2D.

### 5.3 The regulatory effect of melatonin on liver metabolism

Age-related circadian rhythm disorders are associated with the onset of inflammation, and the decline in melatonin levels is closely linked to inflammatory responses in the liver. Research indicates that melatonin binds to G protein-coupled receptors MT1 and MT2 in humans and rodents, and liver cells express melatonin receptors that can be regulated by melatonin (Renzi et al., 2011). Early experiments have shown that melatonin treatment can improve dyslipidemia (Sun et al., 2015). In patients with NAFLD, melatonin (2–5 mg/day) treatment for 14 months significantly reduced triglyceride (TG) and low-density lipoprotein cholesterol (LDL-C) levels (Sun et al., 2016). Melatonin can inhibit the expression of vascular endothelial growth factor (VEGF)-A and transforming growth factor (TGF)-β1 in hepatocytes, thereby providing protective effects against toxins or carcinogens (such as CCl_4_) that cause liver fibrosis (Bona et al., 2018). Therefore, melatonin has been identified as a potential candidate for the treatment of liver disease.

The hepatoprotective effects of melatonin have been confirmed in various metabolic disease models. The α2-HS glycoprotein gene (AHSG) and its protein fetuin-A (FETUA) are one of the liver-derived factors known to be associated with insulin resistance and type 2 diabetes. Heo et al.'s study showed that in HepG2 cells treated with palmitic acid, phosphorylated AKT expression decreased, while FETUA expression increased. Melatonin attenuated FETUA expression, improving insulin resistance and hepatic steatosis (Luo et al., 2020; Wang L. et al., 2022). Additionally, melatonin reduced the expression of endoplasmic reticulum stress markers (CHOP, BiP, ATF-6, XBP-1, ATF-4, and PERK) induced by palmitic acid and alleviated hepatic steatosis. This suggests that melatonin treatment can reduce FETUA expression and endoplasmic reticulum stress marker levels in the liver and serum of obese mice (Luo et al., 2020). In summary, melatonin improves hepatic insulin resistance and hepatic steatosis. These findings suggest that melatonin plays an important role in maintaining hepatic metabolic function and preventing non-alcoholic fatty liver disease.

### 5.4 Regulatory mechanisms of melatonin on fat tissue metabolism

Melatonin regulates adipose tissue function across multiple levels, from adipocyte differentiation to circadian rhythm regulation of energy expenditure. Numerous studies have shown that melatonin can reduce body weight in mice and improve obesity symptoms in mice fed a high-fat diet (Xu et al., 2022). Melatonin tips the energy balance in the direction of reducing food intake and increasing brown adipose tissue energy expenditure, preventing excessive body weight gain (Amaral et al., 2019). In terms of adipose tissue regulation, melatonin has been found to increase demethylation of m6A RNA in adipocytes and inhibit resistin production, thereby improving steatosis (Rong et al., 2019). In white adipose tissue of mice fed a high-fat diet (HFD), multiple pathways associated with inflammatory responses are upregulated, including several interleukin (IL) and tumor necrosis factor (TNF) family genes. Melatonin supplementation leads to reduced levels of IL-1β and TNF-α in white adipose tissue of mice fed a high-fat diet. These findings indicate that IL-1β and TNF-α levels are more significantly elevated in aged mice fed a high-fat diet, suggesting enhanced sensitivity to caloric excess in aged animals. Melatonin alleviates inflammatory responses in white adipose tissue (Lv et al., 2024).

Aging and obesity lead to decreased melatonin levels, downregulation of the PINK1/PARKIN pathway, and reduced mitochondrial autophagy, thereby promoting oxidative stress. Additionally, melatonin regulates CEBPB to activate inflammatory pathways, thereby exacerbating inflammation (Lv et al., 2024). Given that mitochondria are a key site for melatonin synthesis and that melatonin promotes mitochondrial autophagy (Reiter et al., 2024; Yang et al., 2024), this enhances the clearance of damaged mitochondria, thereby maintaining cellular health (Cao et al., 2024). Studies indicate that melatonin activates mitochondrial autophagy through pathways such as PINK1/PARKIN. This suggests that melatonin treatment increases mitochondrial autophagy in white fat, supporting the importance of mitochondrial quality control in fat tissue health and highlighting the role of autophagy-related pathways in mediating the beneficial effects of melatonin.

### 5.5 Effects of melatonin on skeletal muscle metabolism

Previous studies have confirmed the benefits of melatonin for ageing skeletal muscle. In a preliminary study by Sayed et al. (2016), using C57BL/6J mice of different ages (3 months, 12 months, and 24 months), early onset of sarcopenia was observed at 12 months. This was characterized by a decline in physical activity and muscle mass, accompanied by an increase in the frailty index (FI). Additionally, changes in muscle structure and ultrastructure were observed, including reduced size and loss of type II muscle fibers, as well as increased mitochondrial size, suggesting alterations in mitochondrial dynamics. These changes worsened further in aged animals. Melatonin treatment improved muscle function and structure in aged mice while reducing mitochondrial and apoptotic nuclear damage in muscle tissue. Therefore, melatonin is suggested as a potential treatment for sarcopenia (Sayed et al., 2018). Melatonin can counteract circadian rhythm disruption, chronic inflammation, oxidative stress, mitochondrial damage, and muscle mass loss in aged muscles (Sayed et al., 2021).

The absence of melatonin results in a reduced total amount of GLUT4 in all of the insulin-sensitive tissues (white and brown adipose tissue, skeletal and cardiac muscle) and impaired central and peripheral insulin signaling (Cipolla-Neto and Amaral, 2018). Watanabe et al.'s study in a goldfish model provided unique insights, finding that melatonin increases brain glucose uptake through a non-insulin-dependent mechanism at night. In fasting individuals, nighttime plasma melatonin levels were significantly elevated, while insulin levels were significantly reduced. Additionally, glucose uptake in the brain, liver, and muscle tissues also significantly increases at night (Watanabe et al., 2023). This finding is of great significance for understanding the role of melatonin in maintaining nocturnal glucose homeostasis. Melatonin also inhibits cell apoptosis by regulating the BAX/BCL2 balance and reducing caspase-3 activity and expression, thereby playing a crucial role in cell apoptosis (Mehrzadi et al., 2021).

### 5.6 Effects of melatonin on bone metabolism

There is a strong association between the circadian rhythm system, melatonin, and bone health markers. Elevated levels of biochemical markers of bone resorption (i.e., collagen type I amino-terminal cross-linking peptide [NTX], collagen type I C-terminal peptide [CTX]) and, to a lesser extent, bone formation (osteocalcin, P1NP) during the hours of darkness when melatonin levels are highest suggest a strong association between the sleep/wake cycle and melatonin rhythms; when sleep and circadian rhythms are disturbed, bone remodeling processes are disrupted and have been shown to be induced by melatonin (Munmun and Witt-Enderby, 2021). Melatonin levels decline progressively with age and after menopause, which may be associated with the development of osteoporosis in the elderly and may contribute to the onset of bone loss in peri- and postmenopausal women. The decline in melatonin levels with age is associated with a decrease in bone-protective hormones (e.g., oestrogen, progesterone, and testosterone) (Al-Azzawi and Palacios, 2009) and an increase in bone-resorbing hormones (e.g., cortisol) (Yiallouris et al., 2019), which may lead to an imbalance in bone remodelling and progressive bone loss (Munmun and Witt-Enderby, 2021).

As we age and our lifestyles become disrupted (chronic jet lag, social jet lag, shift work), increased levels of reactive oxygen species (ROS) in the body can inhibit osteoblast formation, induce osteoclast formation, and gradually lead to low bone density, bone loss, age-related osteoporosis, or osteoporosis-related fractures (Mody et al., 2001; Maria and Witt-Enderby, 2014). Melatonin is a powerful antioxidant and free radical scavenger that can also prevent bone loss by inhibiting these systems and subsequently softening RANKL levels, thereby re-establishing a healthy balance between RANKL and OPG ratios (Munmun and Witt-Enderby, 2021). Strategies involving the administration of melatonin as an alternative therapy to restore normal nighttime peaks or therapeutic levels exceeding nighttime peaks may be used to prevent or slow bone loss or reverse bone loss.

Melatonin has been found to stimulate Per2, with Per2 levels peaking during the dark period when melatonin levels are highest. Glucocorticoids and their homologous receptors, as well as heat shock proteins 70 and 90, also regulate bone rhythms by influencing bone tissue reactivity through the rhythmic inhibition of glucocorticoid receptors (Zvonic et al., 2007; Bedrosian et al., 2016; Munmun and Witt-Enderby, 2021). The role of melatonin as a circadian rhythm molecule may also influence the regulation of aging rhythms in mice. Additionally, the rhythmic patterns of each tissue may vary, necessitating further investigation into the rhythmic behavior of melatonin within each tissue.

### 5.7 The therapeutic potential and clinical application prospects of melatonin

As mentioned earlier, melatonin possesses potential therapeutic properties and is highly useful in treating various diseases. This is primarily attributed to its antioxidant and anti-inflammatory effects. Since melatonin plays a role in regulating circadian rhythms, melatonin supplementation has demonstrated promising results in improving sleep quality and metabolic function in the elderly. Exogenous administration of melatonin improves non-restorative sleep as well as the amplitude and misalignment of circadian rhythms (Zisapel, 2018). Additionally, melatonin replacement therapy can restore sleep-wake cycle control in elderly individuals with neurodevelopmental disorders and children with insomnia (Wade et al., 2014).

Melatonin also benefits neuronal development and can therefore be used to treat autism spectrum disorders (ASD). Children with ASD who supplemented with melatonin for approximately 3 months not only experienced improved sleep quality and reduced sleep latency but also showed improvements in behavioural problems (Kvetnoy et al., 2022), which is beneficial for children with chronic sleep-wake cycle disorders (Zisapel, 2018). Melatonin and its agonists have been found to improve sleep quality and neuroprotection in patients with Huntington’s disease (Cuomo et al., 2017). This suggests that melatonin may have a significant role in improving patients’ quality of life and reducing disease progression. Individualised melatonin therapy requires consideration of multiple factors, as the impact of melatonin on metabolic control may depend on several factors, including the timing and duration of use, as well as individual genetic variations. Therefore, future treatment protocols should consider the influence of factors such as MTNR1B gene polymorphisms, age, gender, and disease status on treatment response. Melatonin lack of toxicity and clinical safety were also shown in studies (Sánchez-Barceló et al., 2010) involving adults and elder patients who were treated with various doses (0.1–300 mg/d) of oral, intravenous, or rectal suppository melatonin for short or long treatment of sleep disorders, jet lag, depressive disorders, attention-deficit/hyperactivity disorder, autism, amyotrophic lateral sclerosis, Huntington disease, diabetes and metabolic syndrome, polycystic ovary syndrome, and frailty, among others (Cipolla-Neto and Amaral, 2018).

## 6 Summary

**FIGURE 6:**
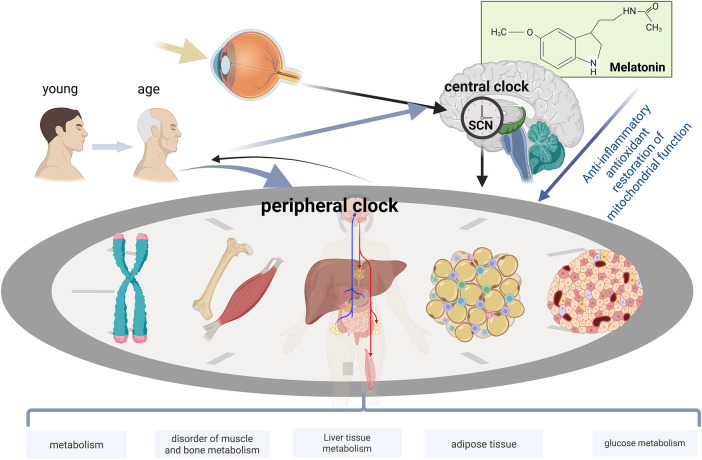
The systemic effects of aging on the circadian rhythm-metabolic networks of multiple organ systems. Aging modulates neural and hormonal signals through the central representative SCN to coordinate peripheral clocks. Age-related decline in clock gene expression and metabolic function is observed in each peripheral tissue (pancreas, liver, adipose tissue, skeletal muscle, and bone). Melatonin supplementation exerts regulatory effects in multiple organs with disrupted circadian rhythms, including the pancreas, liver, adipose tissue, skeletal muscle, and bone.

With aging, the regular circadian rhythm gradually deteriorates, potentially triggering various metabolic disorders and elevating the risk of multiple diseases. The normal functioning of the circadian rhythm is crucial for insulin secretion, hepatic lipid synthesis, and glucose-lipid metabolism processes. The structure and function of muscles and bones depend on precise circadian rhythm regulation, and age-related circadian rhythm disorders can impair these tissues, leading to muscle and bone degeneration. Overall, circadian rhythm disorders have become a key pathophysiological mechanism underlying a range of age-related metabolic dysfunctions and diseases. Melatonin serves as a key messenger molecule linking the central circadian rhythm to peripheral metabolism. With aging, melatonin secretion declines, forming a “melatonin deficiency-accelerated aging” vicious cycle, and exerts regulatory effects on multiple organs, including the pancreas, liver, adipose tissue, skeletal muscle, and bones, through diverse mechanisms such as antioxidant, anti-inflammatory, and mitochondrial protective functions. Melatonin holds promise as an important therapeutic strategy for slowing aging and improving metabolic health. A clear understanding of this mechanism is crucial for developing strategies to delay aging and manage metabolic diseases (Figure 6).

## 7 Limitations and future directions

Current understanding of the interaction between circadian rhythms and aging has the following limitations: Most mechanistic studies rely primarily on rodent models, which may not fully replicate the complexity of human circadian rhythm physiology. Compared to laboratory mice, humans exhibit distinct activity patterns, longer lifespans, and unique metabolic characteristics, particularly significant differences in the amplitude and phase of metabolic rhythms. These differences make it challenging to effectively model human rhythms in the highly homogeneous populations of laboratory animals, thereby limiting the generalizability of research findings. Additionally, technical and ethical challenges in conducting long-term longitudinal studies limit our comprehensive understanding of age-related trajectories of circadian rhythms, while molecular-level circadian rhythm detection technologies still face cost and technical barriers in clinical applications.

The following promising research directions warrant further exploration: (1) At the precision medicine level, establish individualized circadian rhythm phenotype prediction models by integrating multi-omics data, and develop time-based therapeutic strategies tailored to individual biological clock characteristics. (2) In terms of technological innovation, utilize wearable devices and artificial intelligence technologies to achieve continuous monitoring and intelligent analysis of circadian rhythms, thereby providing real-time data support for personalized interventions. (3) In terms of biomarkers, identify biomarkers for circadian rhythm disorders to facilitate early detection and intervention. (4) In terms of combined interventions, investigate the synergistic effects of circadian rhythm-targeted interventions (e.g., melatonin, phototherapy) with traditional metabolic therapies; explore combined interventions involving nutritional chronotherapy, exercise chronotherapy, and drug therapy; and develop multi-modal intervention protocols to maximize treatment efficacy. (5) Longitudinal studies: Conduct long-term cohort studies to track changes in circadian rhythm function throughout the human lifespan, aiming to better understand the spatiotemporal progression of circadian rhythm decline and establish circadian rhythm reference standards for different populations.
